# Blackthorn Flower Extract Impact on Glycaemic Homeostasis in Normoglycaemic and Alloxan-Induced Hyperglycaemic C57BL/6 Mice

**DOI:** 10.17113/ftb.59.03.21.7057

**Published:** 2021-09

**Authors:** Irena Crnić, Tajana Frančić, Petar Dragičević, Vedran Balta, Verica Dragović-Uzelac, Domagoj Đikić, Irena Landeka Jurčević

**Affiliations:** 1 Faculty of Food Technology and Biotechnology, University of Zagreb, Pierottijeva 6, 10000 Zagreb, Croatia; 2 School of Medicine, University of Zagreb, Šalata 3, 10000 Zagreb, Croatia; 3 Faculty of Science, University of Zagreb, Rooseveltov trg 6, 10000 Zagreb, Croatia

**Keywords:** hyperglycaemia, blackthorn (*Prunus spinosa* L.) flower extract, oral glucose tolerance test, insulin, α-amylase

## Abstract

**Research background:**

The use of plants and their extracts in treatments of chronic diseases is widely known in traditional medicine. The aim of this study is to determine the effects of 10-day consumption of blackthorn (*Prunus spinosa* L.) flower extract on blood glucose, glycaemic load, serum α-amlyase activity and insulin concentration in normoglycaemic and hyperglycaemic (alloxan-induced) mice model.

**Experimental approach:**

Normoglycaemic and hyperglycaemic (treated with alloxan, 150 mg per kg body mass) C57BL/6 mice were administered daily, during 10 days, blackthorn flower extract by gavage. The sugar mass concentration within the extract was determined by HPLC analysis. In mice, blood and serum blood glucose concentrations, and oral glucose tolerance test were determined by blood glucometer. Serum insulin concentration was determined by ELISA assay and α-amylase activity by colourimetric assay.

**Results and conclusions:**

The blackthorn flower extract increased glucose concentrations in normoglycaemic mice by 30% after the 1st and 5th day and by 17% after the 10th day of consumption. It is a consequence of released sugars because sugar analysis revealed 59.8 mg/L monosaccharides, mainly fructose (55.7 mg/L) and glucose (24.3 mg/L) in the extract. On the contrary, the extract consumption reduced serum blood glucose in hyperglycaemic mice by 29% after 10 days of treatment. Oral glucose tolerance test also confirmed that in the hyperglycaemic group treated with blackthorn flower extract glucose homeostasis was improved and showed decrease in blood glucose. Serum insulin concentration increased by 49% and serum α-amylase activity by 46% after 10 days of treatment with blackthorn flower extract in hyperglycaemic group. Thus, it can be concluded that blackthorn flower extract improved glucose tolerance, enhanced insulin secretion and lowered serum α-amylase activity.

**Novelty and scientific contribution:**

The obtained results show for the first time the potential of blackthorn (*Prunus spinosa* L.) flower extract in hyperglycaemia management.

## INTRODUCTION

Diabetes mellitus is a chronic metabolic disease that is characterised by chronic impaired blood glucose levels and hyperglycaemia as a result of compromised insulin secretion or impaired insulin action (*1*). Physiologically and biochemically, in various tissues, the chronic hyperglycaemia can induce oxidative stress that terminate normal biological activities and cause a cascade of chronic diabetic complications (*2*).

The usage of plants and their extracts in many treatments of chronic diseases is widely known in traditional medicine, but it also has a big potential in the treatment of hyperglycaemia and diabetic complications, especially by reducing the levels of oxidative stress on a cellular cytoplasmic level (*1*, *3*-*6*). Recent scientific data support the fact that polyphenols including flavonoids, phenolic acids, lignans and stilbenes found in most plants can have a positive effect on many chronic diseases, and act like potent antioxidants and anti-inflammatory agents (*4*, *5*, *7*, *8*).

Blackthorn (*Prunus spinosa* L.) is a rich source of phytochemicals, polyphenols, including phenolic acids and flavonoids, A-type proanthocyanidins, anthocyanins, flavanols and flavones, flavan-3-ols and has many anti-inflammatory, diuretic, blood purifying, spasmolytic and antitumour activities (*3*, *9*-*11*). All these plant phenolic compounds have potent antioxidant capacity (*11*) and there is evidence that they can regulate hyperglycaemic levels, adipocytokine gene expression and enhance some metabolic activities (*12*). The hypoglycaemic effects of medicinal herbs on hyperglycaemia include physiological mechanisms such as enhancing peripheral tissue insulin sensitivity, inhibition of digestive enzymes involved in carbohydrate breakdown and inhibition of glucose absorption in gastrointestinal tract (*13*, *14*). The aim of this study is to determine the effect of 10-day consumption of blackthorn flower extract on blood glucose, glycaemic load and glycaemic parameters, serum α-amlyase activity and serum insulin concentration in alloxan-induced hyperglycaemic mice. Despite the traditional use of blackthorn as medicinal herb, there are no studies that investigated the effect of its flower extract on glycaemic homeostasis. The obtained results might serve as a guide towards the design of nutraceutical polyphenol mixture as supportive therapy in the hyperglycaemia treatment.

## MATERIALS AND METHODS

### Study design in vivo: animals and diets

For this experiment, a total of 24 male inbred C57BL/6 mice weighing (30±1.5) g were obtained from the Department of Animal Physiology, Faculty of Science, University of Zagreb, Croatia. Animals had access to a standard laboratory diet and tap water *ad libitum* and received 12 h of light per day. The standardized diet was 4 RF 21 (Mucedola srl, Settimo Milanese, Italy). The composition of the standardised pellet mouse feed included wheat, wheat straw, hazelnut skins, maize, soya bean, corn gluten feed, fishmeal, dicalcium phosphate, sodium chloride, whey powder, soya bean oil, yeast, and components and supplements: 12% moisture, 18.5% protein, 3% fats, 6% crude fibre, 7% crude ash, E672 (vitamin A), E671 (vitamin E), E1 (Fe), E2 (I), E3 (Co), E4 (Cu), E5 (Mn) and E6 (Zn). Maintenance and care of all experimental animals was performed according to the guidelines of the Republic of Croatia (*15*). The experimental procedures were approved by the Bioethics Committee of the Faculty of Science, University of Zagreb, Zagreb, Croatia (*16*) and were conducted according to the Guidelines for *in vivo* experiments and accepted international standards (*17*).

### The treatment of the animals and experimental design

**Fig. 1:**
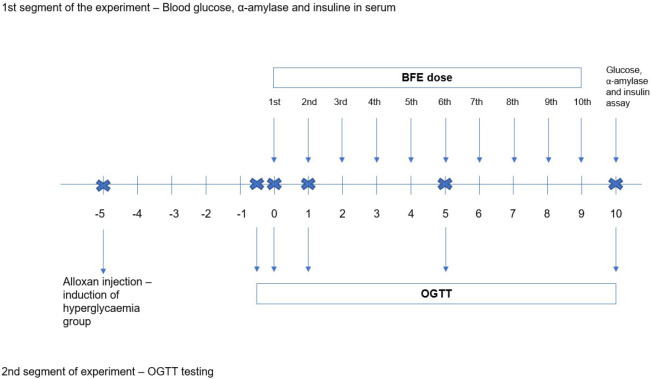
The experimental design. The experiment consisted of two segments: 1st glucose, insulin and α-amylase measurements on the 1st, 5th and 10th day of treatment with blackthorn (*Prunus spinosa* L.) flower extract (*w*(BFE)=25 mg/kg) and 2nd the glucose challenge of treatment groups (oral glucose tolerance test (OGTT)) and determination of glucose concentration within 120 min after glucose and extract intake on the 1st, 5th and 10th experimental days. The baseline group in OGTT gives the glucose dynamics within 20 min in experimental groups without the glucose intake and day 0 is the glucose challenge in experimental groups prior to treatment with blackthorn flower extract. Day 1 is glucose challenge 24 h after the flower extract treatment (single dose) and days 5 and 10 are the glucose challenge (OGTT) after five and ten repeated flower extract doses

Animals were randomly divided according to the treatment in four groups (Fig. 1). The first group, control (C), is normoglycaemic and untreated, receiving the phosphate-buffered saline (PBS, Sigma-Aldrich Chemie GmbH, Merck, Darmstadt, Germany) instead of the extract. The second group is a normoglycaemic group treated with the blackthorn flower extract. Furthermore, the third, hyperglycaemic group, is treated with alloxan (AL). The fourth is the hyperglycaemic group treated with alloxan and blackthorn flower extract (AL+PSE). Each group contained 6 animals.

The experiment consisted of two connected segments involving the monitoring of blood glucose concentrations (Fig. 1). The first part of the experiment analysed the changes of the blood glucose and insulin concentration and amylase activity after a single (24 h), five and ten blackthorn flower extract doses to determine the changes within these parameters over the period of 10 days. The second part of the experiment consisted of the oral glucose tolerance test (OGTT) on the same experimental days, with the aim to establish the dynamics of physiological blood glucose management after oral glucose addition in the experimental groups over the time course of 120 min.

#### Induction of experimental hyperglycaemia with alloxan

To pharmacologically create chronic hyperglycaemia in the animals, the animals were treated with alloxan (Sigma-Aldrich, Merck, St. Louis, MO, USA) at a mass fraction of 150 mg per kg of body mass, intraperitoneally with a syringe, five days prior to the beginning of the experimental treatments of groups with blackthorn flower extract (Fig. 1). Twenty-four hours after the alloxan injection, blood glucose concentration of mice was assessed and mice with >8.5 mmol/L for 5 days were considered to have hyperglycaemia. After 5 consecutive days of hyperglycaemia this group of mice was treated with blackthorn flower extract on the same time (day 0) as the normoglycaemic groups (except control group which was treated with PBS).

### Treatment with blackthorn flower extract

The treatments (control with PBS and normoglycaemic and hyperglycaemic groups with blackthorn extract) were administered as single daily oral gavage doses in a volume of 0.2 mL. The groups treated with the extract were dosed with 25 mg of total phenolics in the extract solution per kg of body mass of the animal. The preparation, analytical details and chemical compositions of extracts determined by ultraperformance liquid chromatography-tandem mass spectrometry (UPLC-MS/MS) were published previously (*18*) where it was shown that the extract contained different types of polyphenols including phenolic acids, flavan-3-ols, flavones and especially flavonols (kaempferol and quercetin glycosides) together with the quantities present in blackthorn dry flower and the administered blackthorn extract solution. Prior to the administration of the desired doses, the original extract was evaporated under reduced pressure at 45 °C to concentrate the solution of polyphenols and to remove excess alcohol. This reduced the amount of alcohol to minimum to be safe for mice. The concentrated solution was re-dissolved and further diluted with water to achieve the final mass fraction of total phenolics, expressed as gallic acid equivalents, of 25 mg/kg, which were administered daily as 0.2 mL of gavage volume to C57BL/6 mouse. Our previous study (*19*), where we applied the same dose but in different regime (single dose, 24 h, plasma bioavailability), gives a detailed list of components and mass fractions of individual polyphenolics in the extract consumed by animals as well as the analysis of phenolic content in food pellet (regular food that both groups consumed as normal chow). The polyphenolic content was assessed by UPLC-MS/MS analysis in commercial Mucedola food pellets since the control group was fed pellets *ad libitum* (average 3500-4000 mg pellets per mouse per day). The same types of polyphenols were determined in pellets, but the total phenolic content (as the sum of all determined polyphenolic compounds) was negligible (4.64 μg per 100 mg dry mass of pellet). A dominant polyphenolic compound that comprised almost 54% of total phenolic content within the feed pellets was flavonol isorhamethin-rutinoside (2.50 μg/100 mg), while other compounds were determined in the range from 0.005 to 0.175 μg/100 mg or undetected.

### Determination of sugar mass concentration in blackthorn flower extract

The sugars were simultaneously analysed by a direct injection of the blackthorn extracts, previously filtered through a 0.45-µm pore size membrane filter (Macherey-Nagel GmbH & Co. KG, Düren, Germany). Chromatographic separation was performed using HPLC analysis with Agilent 1260 quaternary LC Infinity system (Agilent Technologies, Santa Clara, CA, USA) equipped with refractive index (RI) detector, an automatic injector and ChemStation software. Sugars (fructose, glucose and sucrose) were separated on a Cosmosil Sugar-D (250 mm×4.6 mm i.d.) column (Nacalai Tesque, Inc., Kyoto, Japan). The solvent composition and the used gradient conditions were described previously by Bogdanov and Baumann (*20*). For isocratic elution, mobile phase A contained 80% acetonitrile in water. Operating conditions were: constant flow rate 1.3 mL/min in 20 min, column temperature 30 °C, injection volume 10 µL and equilibration time 2 min. Detection was performed with refractive index detector. Identification of sugars was carried out by comparing retention times of the authentic standards (fructose, glucose and sucrose). The quantifications of sugars were made by the external standard method. All sugar standards were dissolved in methanol at a concentration of 50 mg/L. Working sugar standard solutions were prepared by diluting the initial solution to yield mass concentrations in a range from 1 to 50 mg/L. Quantitative determination was carried out using the calibration curves of the standards:

fructose: y=70684x+4030, R^2^=0.99 /1/

glucose: y=72170x+6655.3, R^2^=0.99 /2/

sucrose: y=71630x+1199.3, R^2^=0.00 /3/

### Collection of blood samples from the animals

Blood samples were collected from the tail vein into EDTA tubes that were used for insulin and amylase assay, while a drop of whole blood was used for blood glucose assay. The blood samples for insulin and amylase assay were mixed thoroughly to prevent blood clotting and were centrifuged at 2000×*g* for 10 min in Hettich® centrifuge Mikro 200R (Merck, Darmstadt, Germany).

### Determination of glucose concentration in blood samples

The glucose concentration in whole blood samples (a drop of whole blood) from the mice tail vein was determined by blood glucometer that uses test strips to assess a glucose oxidoreductase-mediated dye reaction, according to the manufacturer’s instructions (MediSmart® Sapphire blood glucose system, Lobeck Medical AG, Frick, Switzerland). The blood glucose concentrations were measured on the 1st, 5th and 10th day and the same assay was applied for glucose determination in oral glucose tolerance test (OGTT).

### Determination of insulin concentrations in blood samples

Insulin was determined with Mouse INS ELISA kit according to the manufacturer’s protocol (*21*), which uses the sandwich-ELISA principle. The micro ELISA plate provided in this kit was pre-coated with an antibody specific for mouse INS. Standards or samples were added to the micro ELISA plate wells combined with the specific antibody. Then a biotinylated detection antibody specific for mouse INS and avidin-horseradish peroxidase (HRP) conjugate was added to each well. Only those wells that contained mouse INS, biotinylated detection antibody and avidin-HRP conjugate appeared blue in colour. The enzyme-substrate reaction was terminated by the addition of stop solution and the colour turned yellow. The absorbance was measured spectrophotometrically (spectrophotometer Biochrom Libra S22; Biochrom Ltd., Cambridge, UK) at a wavelength of (450±2) nm.

### Determination of α-amylase activity in blood samples

Amylase activity was determined by colourimetric assay, using Abcam amylase assay kit, according to the manufacturer’s protocol (*22*). Amylase assay kit detects the activity of α-amylase through a two-step reaction. The α-amylase cleaves the substrate ethylidiene-pNP-G7 to produce smaller fragments that are modified by α-glucosidase, causing the release of a chromophore that can be measured at *A*=405 nm. The assay can detect α-amylase activity as low as 0.2 mU.

### Oral glucose tolerance test

Oral glucose tolerance test (OGTT) evaluates the ability to respond appropriately to a glucose challenge. The animals were fasted 6 h before commencing the experiment. The test was performed by oral administration of glucose load of 2 mg/kg, in all groups. Blood samples were collected from the tail vein at 0, 15, 30, 60 and 120 min after the oral glucose addition. The glucose concentrations were measured using a blood glucose meter (MediSmart® Sapphire blood glucose system) according to the manufacturer’s instruction. Total glycaemic response to OGTT was calculated from the areas under the curve. Baseline blood glucose concentrations were taken before the administration of glucose and baseline measurements were done to establish glucose dynamics in untreated mice in order to evaluate the difference of hyperglycaemic and normoglycaemic groups. Measurement on day 0 was conducted immediately after administrating the blackthorn flower extract solution to establish the potential of the extract to change blood glucose concentration. After day 0 glucose was measured on days 1, 5 and 10 at the same intervals: 0, 15, 30, 60 and 120 min.

### Statistical analysis

All the data were expressed as mean value±standard deviation (S.D.). IBM SPSS Statistics v. 17 (*23*) and GraphPad Prism v. 17.0 (*24*) were used for visualisation of data and statistical comparisons between groups by Kruskal-Wallis test (ANOVA).

## RESULTS AND DISCUSSION

The blackthorn (*Prunus spinosa* L.) flower extract is a rich source of polyphenols, the most abundant being quercetin and kaemferol glycosides, represented by kaempferol-3-O-pentoside and rhamnoside and quercetin-3-O-pentosyl-pentoside, and also phenolic compounds belonging to the classes of hydroxycinnamic acids and flavonol gycosides (*18*).

**Fig. 2:**
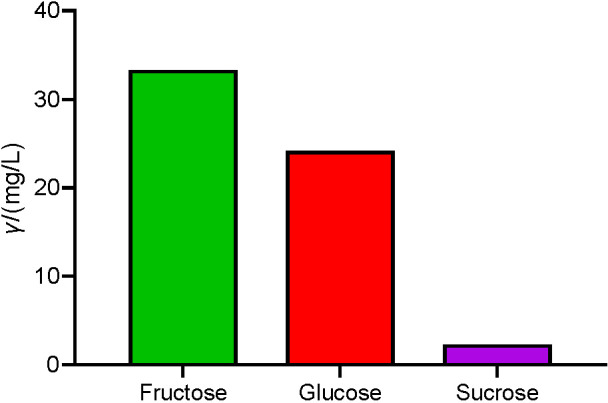
Sugar mass concentration determination in blackthorn (*Prunus spinosa* L.) flower extract

The extract contains a significant concentration of sugar (Fig. 2), accounting for the total of 59.8 mg/L. The analysis of individual sugar types within the extract revealed that the most dominant sugar in blackthorn flower extract was fructose at 55.7% (33.3 mg/L), followed by glucose at 40.3% (24.3 mg/L) and sucrose, with only 3.8% (2.3 mg/L) of total sugar concentration. Therefore, the blackthorn flower extract contained 96% monosaccharides and only 3.8% disaccharides.

**Fig. 3:**
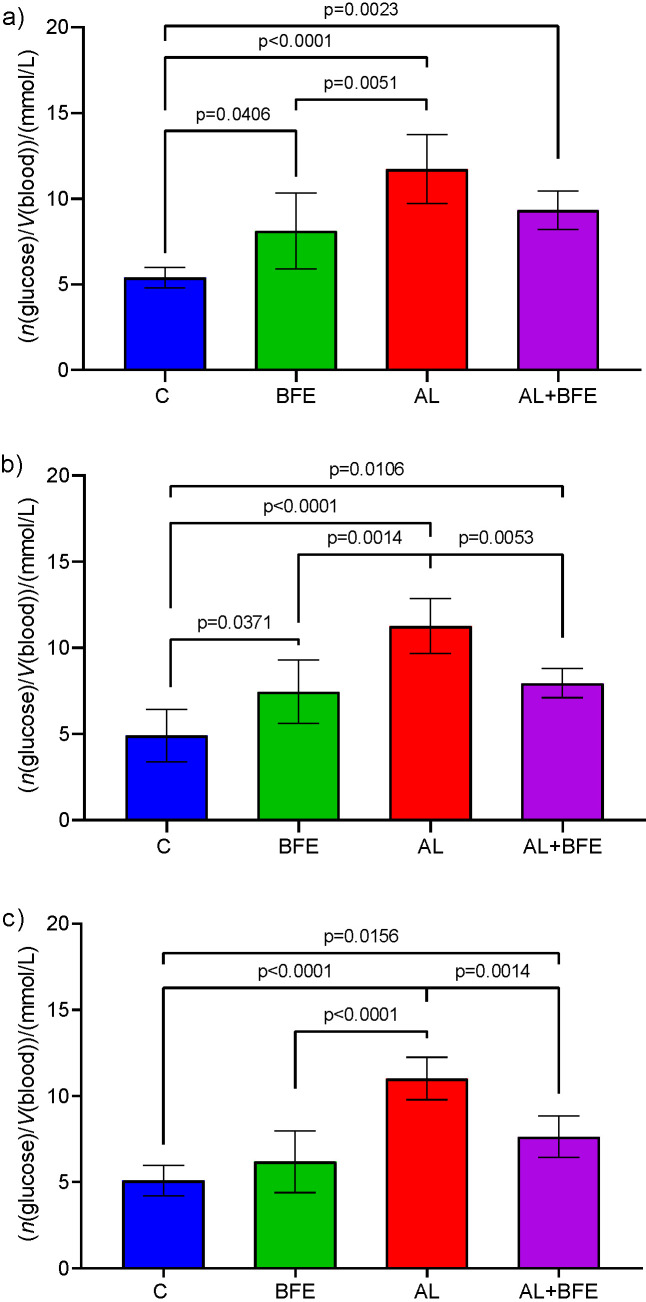
Serum blood glucose concentrations in experimental groups of C57BL/6 mice measured on: a) the 1st experimental day, after single dose, b) on the 5th experimental day, after five daily doses, and c) on the 10th experimental day, after ten consecutive daily treatments with blackthorn (*Prunus spinosa* L.) flower extract (*w*(BFE)=25 mg/kg). The values are mean±S.D. (*N*=6 for each group). Significant difference values are marked with p≤0.05; highly significant difference values are marked with p≤0.001

The results in Fig. 3 show serum blood glucose levels in the experimental groups of C57BL/6 mice and the effects of blackthorn flower extract intake on blood glucose in normoglycaemic and hyperglycaemic (alloxan-induced) mice after a single (1st day) or five (5th day) and ten (10th day) doses. The flower extract intake resulted in slightly but significantly (p≤0.05) increased blood glucose concentration in normoglycaemic mice treated with the extract compared to the untreated control mice, but only on the 1st (Fig. 3a) and 5th (Fig. 3b) experimental day. At the end of the experiment (Fig. 3b) and ten received doses, despite the treatment with the extract, the blood glucose concentrations were the same as in control group without any significant differences between the groups (Fig. 3c).

When the blackthorn flower extract was administered to the hyperglycaemic mice, there was a significant (p≤0.05) reduction of blood sugar concentration (compared to alloxan-treated hyperglycaemic group) after five (Fig. 3b) and ten (Fig. 3c) received extract doses (5th and 10th experimental day, Fig. 3b and Fig. 3c, respectively). This signifies that the consumption of the flower extract in alloxan-treated hyperglycaemic group decreased hyperglycaemia after at least 5 or 10 repeated daily consumptions. Interestingly, the single dose (1st experimental day, Fig. 3a) was not sufficient to reduce the hyperglycaemia significantly (comparison of alloxan-induced group and alloxan-induced hyperglycaemic group treated with the flower extract).

**Fig. 4:**
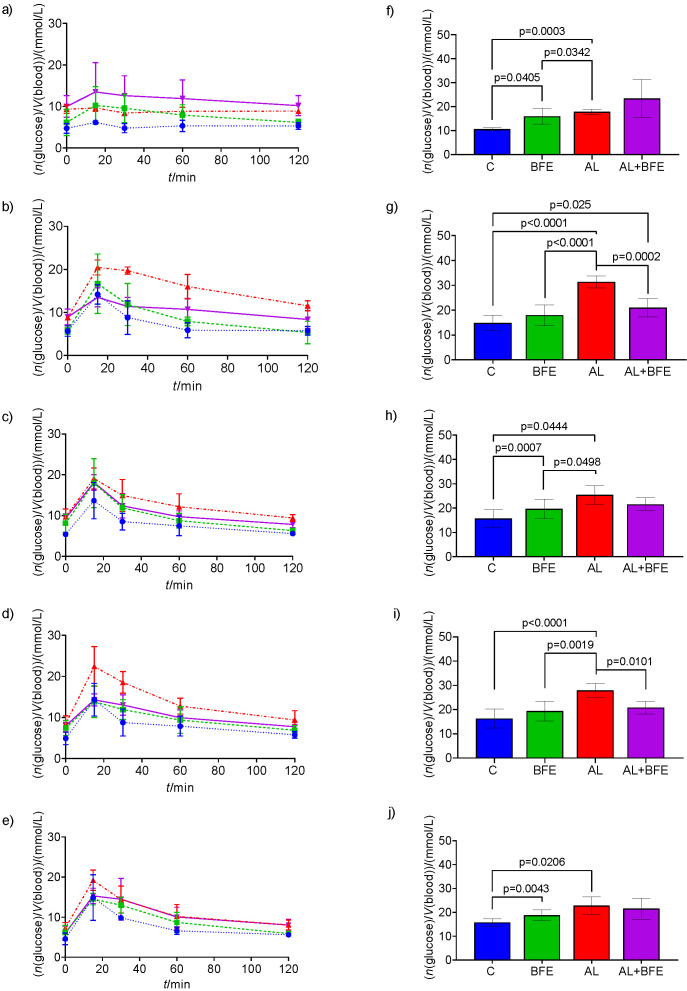
Oral glucose tolerance test (OGTT) in experimental groups of C57BL/6 mice challenged with single oral glucose intake and daily repeated doses of blackthorn (*Prunus spinosa* L.) flower extract (*w*(BFE)=25 mg/kg) treatment. The blood glucose concentration within 2 h: a-e) represents the pharmacokinetic curves of blood glucose in treatment groups, and f-j) shows the area under these curves, where the letters represent the following: a and f) baseline, b and g) day 0, c and h) day 1, d and i) day 5, and e and j) day 10. The values are mean±S.D. (*N*=6 for each group). C=control (blue), BFE=blackthorn extract (green), AL=alloxan (red), AL+BFE=combined treatment with alloxan and BFE. Significant difference values are marked with p≤0.05; highly significant difference values are marked with p≤0.001

The oral glucose tolerance test (OGTT) in Fig. 4 shows the 2-hour dynamics of blood glucose adaptation. The blood glucose concentrations are shown as the pharmacokinetic curves (Figs. 4a-e) in all treatment groups after the consumption of glucose solution. The effects of parallel blackthorn flower extract and glucose intake in normoglycaemic (control and blackthorn flower extract group) and hyperglycaemic mice (treated with alloxan only or alloxan and flower extract) are shown separately on different experimental days. Fig. 4a and Fig. 4f depict the baseline blood glucose concentration without glucose addition in all groups but with a single extract dose in normoglycaemic and hyperglycaemic groups. Fig. 4b and Fig. 4g show treated groups challenged with oral glucose ingestion prior to blackthorn extract treatment. Fig. 4c and Fig. 4h show the groups receiving a single (1st day) dose, Fig. 4d and Fig. 4i those receiving five (5th day) and Fig. 4e and Fig. 4j ten (10th day) flower extract doses. It was noticed (Figs. 4a-e) that combined treatment with alloxan and flower extract improved glucose homeostasis and decreased blood glucose concentration compared to alloxan treatment alone. The differences in the areas under the pharmacokinetic curves were analysed (Fig. 4). For analysis simplicity, statistical significance was calculated only for the values of areas under the curve (Figs. 4f-j), which indicate glucose dynamics after glucose addition, over a period of 120 min for each group. There was a significant difference between the hyperglycaemic group treated with the blackthorn flower extract and the alloxan-treated hyperglycaemic group (p=0.0002) on day 0 (Fig. 4g) and day 5 (Fig. 4i). A significant difference in baseline treatment (p=0.0405) was between the group treated with the extract and the control on days 1 (Fig. 4h) (p=0.0007) and 10 (Fig. 4j) (p=0.004). Cumulatively, the blood glucose concentration over the period of 120 min presented in the graphs of the areas under the curve shows that in flower extract group, glucose homeostasis is achieved faster, even after 10 days of treatment.

**Fig. 5:**
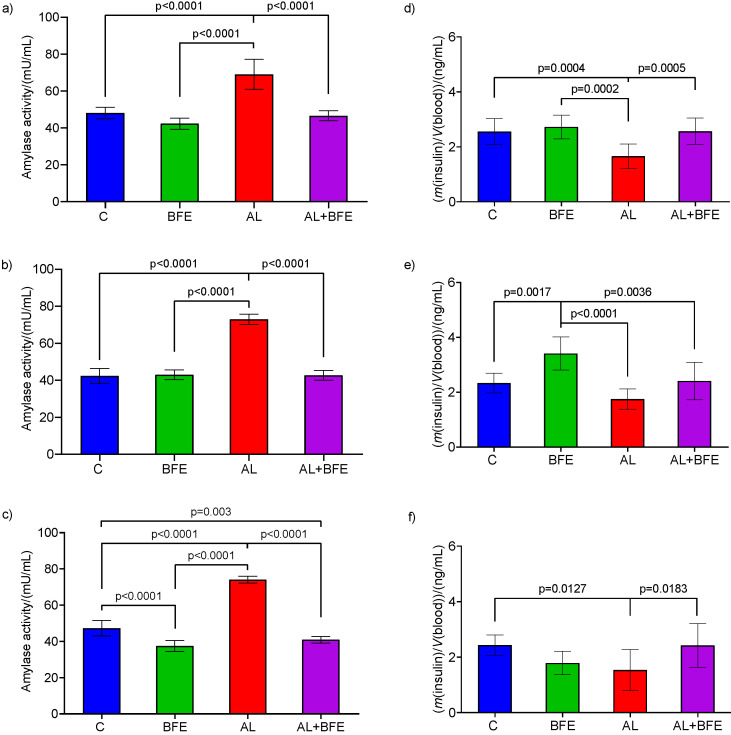
Activity of α-amylase in the serum of C57BL/6 mice on: a) day 1, b) day 5, and c) day 10. Mass concentration of insulin in the serum of C57BL/6 mice on: d) day 1, e) day 5, and f) day 10. The values are mean±S.D. (*N*=6 for each group). Significant difference values are marked with p≤0.05; highly significant difference values are marked with p≤0.001

The results in Figs. 5a-c show concentrations of insulin and activity of α-amylase in the serum of normoglycaemic (control) and hyperglycaemic (alloxan-induced) mouse experimental groups and the effects of blackthorn extract intake (in normoglycaemic and hyperglycaemic group treated with the extract) after single (1st day) or five (5th day) and ten (10th day) repeated doses.

The intake of the flower extract did not affect amylase activity until the 5th experimental day but resulted in significantly lower serum α-amylase activity (p<0.0001) in flower extract group than in the control group until the 10th (Fig. 5c) experimental day. This means that ten repeated doses of daily consumption of blackthorn flower extract had the potential to inhibit amylase activity. Alloxan significantly increased the α-amylase activity in hyperglycaemic group compared to normoglycaemic control group. However, when the extract was administrated to the hyperglycaemic mice, there was a significant reduction in α-amylase activity (p<0.0001) compared to the alloxan-treated group on the 1st (Fig. 5a) and 10th (Fig. 5c) experimental days. The results in Figs. 5d-f show that the concentrations of blood insulin were significantly higher (p=0.0017) on day 5 when the extract was administrated to normoglycaemic mice than in the control group. Alloxan-treated group had a significantly lower insulin concentrations than the control group on the 1st (p=0.0004) (Fig. 5d) and 10th (p=0.013) (Fig. 5f) experimental days. However, when the flower extract was administrated to hyperglycaemic mice, there was a significant increase of serum insulin concentration compared to the alloxan-treated group on the 1st (Fig. 5d) (p=0.0005) and 10th (Fig. 5f) (p=0.018) experimental days.

The consumption of the flower extract, shown with oral glucose tolerance test, both short term (in min) or long term repeatedly during 10 days can modulate blood glucose concentrations and in long term might influence the α-amylase activity and insulin concentrations.

When taken under normoglycaemic conditions (healthy non-hyperglycaemic individuals), the extract shows the ability to slightly but significantly raise blood sugar concentration, as shown in the results for the flower extract group compared to the control group (Fig. 4). In available literature, there are examples of certain plant extracts with similar properties. For example, Tourkey (*25*) reported significantly higher concentrations of glucose in mice treated with *Moringa oleifera* aqueous extract. The observed short-term glucose elevation in normoglycaemia could be explained by significant presence of sugars in the extract. On the other hand, relative normalisation of blood glucose balance in hyperglycaemia could be attributed to richness in polyphenol molecules and to polyphenolic content in the flower extract. Flavonoids are characterised by their basic skeleton arranged in the form C6-C3-C6, two aromatic rings A and B linked by a unit of three carbon atoms. Hydroxyl group and sugars are very common flavonoid skeleton substituents, and they increase the water solubility of flavonoids. Flavonoids exist naturally as glycosides (*26*, *27*). The sugar concentration analysis showed that the flower extract contains a significant concentration of simple sugars, monosaccharides, up to approx. 97% of total sugars. Nevertheless, the results show that in the groups treated with the extract on the 1st and 5th day glucose concentrations tended to be slightly higher than in the control group, although they never reached pathologic hyperglycaemia as in alloxan-induced group. From metabolic and physiologic points of view, nutritional feature of food and beverages that are rich in available carbohydrates is that their consumption induces postprandial hyperglycaemia. The liberated glucose is absorbed within the intestinal enterocytes by the specific transporters (*27*). The fast absorption of glucose activates the regulatory mechanisms of glucose homeostasis. Besides free sugars present in the extract (Fig. 2), some of the sugars that covalently bind to phenolic compounds might be liberated during digestion and absorption, which overall led to the rise in blood serum glucose concentration. It was observed that despite the slight increase of blood glucose concentration under normal metabolic conditions, the extract in hyperglycaemic mice promotes faster recovery and improved glucose homeostasis, as shown in alloxan-induced hyperglycaemic group treated with flower extract, than alloxan treatment alone. Furman *et al.* (*28*) presented a review on medicinal plant extracts that express similar acute hypoglycaemic effect in diabetic rodents, for example *Pycnanthus angolensis* (rich in terpenoids), *Phyllanthus sellowianus* (rich in flavonoids), *Gentiana olivieri* (rich in flavons) and *Cinnamonum zeylanicum* (rich in cinnamaldehyde). Most researchers used polyphenol-rich plant extracts along with other bioactive substances to address their numerous antidiabetic mechanisms, which may include lowering blood glucose, inhibition of polysaccharide digestion, inhibition of hepatic gluconeogenesis, stimulation of peripheral glucose uptake, modulation of intestinal microbiome, antioxidant effect and stimulation of glucagon-like peptide-1 (GLP-1) secretion (*4*, *28*, *29*). Such protective effects are usually ascribed to the richness of phenolic molecules in plant extracts. It is known that blackthorn flowers contain flavonoid complex and flavonol derivates: kaempferol and quercetin with their glycosides bound with arabinose, rhamnose and xylose (*30*). In the flower extract, individual polyphenols like kaempferol and quercetin are glycosylated (*10*). Olszewska and Wolbiś (*10*) found in plant extracts compounds such as kaempferol 3-O-α-l-arabinofuranoside, kaempferol 3-O-(2’’-E-*p*-coumaroyl)-α-l-arabinofuranoside, kaempferol 3-O-β-d-xylopyranoside, kaempferol 7-O-α-l-rhamnopyranoside and kaempferol 3-O-α-l-rhamnopyranoside. In the flower extract we used in this work the composition and concentrations of 28 polyphenols were determined by UPLC-MS/MS (*19*). Flavonol glycosides were found to be the major polyphenolic class in blackthorn flowers, especially quercetin and kaempferol as kaempferol rhamnosyl-hexoside, quercetin-pentosyl-hexoside, kaempferol-pentosyl-hexoside, kaempferol-pentoside, quercetin-rhamnoside, kaempferol-rhamnoside, quercetin acetylhexoside and kaempferol acetylhexoside (*19*). In a previous experiment with a single dose, we show that a significant number of these glycosides can be traced by UPLC-MS/MS method in the plasma of mice (*19*) and in organs such as intestine, liver and kidney (*11*).

Besides the long-term effect of blackthorn flower extract on blood glucose, it is very important to know the short-term dynamics of adaptation of blood glucose after the consumption of glucose solution. The oral glucose tolerance test (OGTT) was used here to evaluate blood glucose homeostasis, intake of blood glucose inside tissues and also indirectly evaluate glucose absorption in 2 h. Fig. 4 shows the blood glucose levels during 2 h. The OGTT evaluates the ability to respond appropriately to a glucose change. For the OGTT, mice are typically fasted overnight, which provokes a catabolic state of metabolism, and reduction of liver glycogen stores. The longer the fast lasts, it decreases the metabolic rate and enhances glucose usage in mice, which is in contrast to humans. As the feeding patterns in mice also do not mimic human behaviour, it may be more physiological to perform an OGTT after a short fasting (*31*). Our results suggest that increased levels of glucose tolerance may be due to increased secretion of insulin, and therefore indicates that the flower extract possesses a hypoglycaemic effect.

In long term hyperglycaemia regulation, the flower extract supplementation in diabetic mice decreased fasting blood glucose concentration and postprandial glucose tolerance, which showed improvement exhibiting similar pattern as normal mice with peak increase at 30 min. Results *in vivo* indicate that the extract displayed a good concentration-dependent inhibitory effect on serum α-amylase activity. Inhibition of α-amylase activities has been demonstrated with polyphenols from plants in many research papers (*4*, *5*, *14*, *32*). The elevated insulin secreting in hyperglycaemic group treated with the extract agrees with *in vivo* results, shown in Fig. 5, and confirms the evidence that the extract acts as a stimulator of insulin secretion.

Some studies suggest that polyphenols from the flower extract reduce hyperglycaemia through various mechanisms. Polyphenols from plants can inhibit α-amylase and α-glucosidase activity, stimulate insulin secretion from the β-cells, balance glucose release from the liver and promote glucose uptake in the insulin-sensitive tissue (*29*). Yang and Kang (*33*) reported that combined treatment of quercetin and resveratrol in streptozotocin-induced diabetic rats maintained the activities of hepatic glucose metabolic enzymes and structure of pancreatic β-cells, and significantly decreased glucose concentration. Consumption of tea (*Camellia sinesis* L.) and coffee (*Coffea arabica* L.) has been related to a lower risk of hyperglycaemia by improvement of glucose tolerance, insulin sensitivity and insulin secretion, reduction of glucose intestinal uptake and regulation of glycaemic homeostasis (*4*). Furman *et al.* (*28*) reported the antihyperglycaemic effect of caffeic acid in diabetic mice by reduction of blood glucose and increased plasma insulin. Chukwuma *et al.* (*5*) reported that combined acute treatment *in vivo* with chlorogenic and caffeic acids improved glucose tolerance as well as plant extracts that contain equal amounts of both phenolic acids, suggesting their synergistic effect. Previous studies of other plants used similar therapeutic approaches for lowering blood glucose and enhancing pancreatic β-cell function, peripheral tissue insulin sensitivity and inhibition of digestive enzymes involved in carbohydrate metabolism.

Our results confirm that the blackthorn flower extract has similar antihyperglycaemic effects as described by Ben Salem *et al.* (*6*), who suggest that ethanol extract of *Cynara scolymus* significantly decreased (p<0.001) the α-amylase activity in serum of diabetic rats, reduced blood glucose rate in the treated group compared to diabetic rats after 28 days of treatment. Similar results were obtained by Tang *et al.* (*34*), who showed that polyphenols from *Punica granatum* L. flower improved insulin sensitivity in diabetic rats, confirmed by OGTT and insulin tolerance tests.

Varshney *et al.* (*35*) showed *in vivo* that tested flavonoids quercetin, rutin, myricetin and kaempferol (all applied in doses of 25 mg per kg of body mass per day) significantly enhanced insulin sensitivity in streptozotocin-induced diabetic mice by decreasing glucose concentration with increase in time. Kaempferol has a highest influence on improving blood glucose level, glucose tolerance and insulin sensitivity. Alkhalidy *et al.* (*36*) reported that after four weeks of oral administration of kaempferol fasting blood glucose concentrations were reduced and insulin sensitivity improved in diet-induced obese C57BL/6 mice.

## CONCLUSIONS

The obtained results show that consumption of blackthorn (*Prunus spinosa* L.) flower extract, measured as oral glucose tolerance test, both short term (in min) or long term (during 10 days) has the ability to slightly but significantly increase blood sugar concentration in metabolically healthy (normoglycaemic) mice. The conclusion of this study is that the intake of blackthorn flower extract for 10 days has a potentially protective effect against hyperglycaemia in C57BL/6 mice by lowering blood glucose concentration, improving glucose tolerance, enhancing insulin secretion and inhibiting serum α-amylase activity. Based on the current investigations, blackthorn flower extract may be a useful support in hyperglycaemia management, including its complications.
